# How Healthcare Professionals in Cardiac Care Address Depressive Symptoms: Experiences of Patients With Cardiovascular Disease

**DOI:** 10.1097/JCN.0000000000000669

**Published:** 2020-02-21

**Authors:** Mats Westas, Johan Lundgren, Ghassan Mourad, Margit Neher, Peter Johansson

**Affiliations:** **Mats Westas, RN** Department of Health, Medicine and Caring Science, Linköping University, Sweden.; **Johan Lundgren, PhD** Department of Health, Medicine and Caring Science, Linköping University, Sweden.; **Ghassan Mourad, PhD** Department of Health, Medicine and Caring Science, Linköping University, Sweden.; **Margit Neher, PhD** Department of Medical and Health Sciences, Linköping University, Sweden.; **Peter Johansson, PhD** Department of Health, Medicine and Caring Science, and Internal Medicine, Linköping University, Norrkoping, Sweden.

**Keywords:** cardiovascular disease, cardiovascular nursing, depressive symptoms, qualitative research

## Abstract

Depressive symptoms are common in patients with cardiovascular disease (CVD) and are associated with a poorer quality of life and prognosis. Despite the high prevalence and negative consequences, the recognition of depressive symptoms is low. More knowledge about patients' perceptions of how depressive symptoms are addressed by healthcare professionals is therefore needed.

**Objectives:**

The aim of this study was to explore the experiences of patients with CVD of how healthcare professionals address and manage depressive symptoms in clinical cardiac care encounters.

**Methods:**

A qualitative, semistructured interview study was performed. Data were analyzed using inductive thematic analysis.

**Results:**

In total, 20 patients with CVD previously treated for depressive symptoms were included (mean age, 62 [range, 34–79] years; 45% women). Three main themes emerged: (1) “not being seen as a whole person,” (2) “denying depressive symptoms,” and (3) “being provided with help.” The patients perceived that healthcare professionals mainly focused on somatic symptoms and disregarded their need for help for depressive symptoms when patients raised the issue. Some patients stated that they received help for depressive symptoms, but this depended on the patients' own ability to communicate their needs and/or having social support that could alert them to the importance of doing so. Patients also described that they downplayed the burden of depressive symptoms and/or did not recognize themselves as having depressive symptoms.

**Conclusion:**

Depressive symptoms were overlooked in patients with CVD, and psychological needs had not been met. A good ability to address needs and having good social support were useful for receiving help with depressive symptoms.

In patients with cardiovascular disease (CVD), including heart failure (HF), atrial fibrillation, myocardial infarction, and angina pectoris (ie, ischemic heart disease), depressive symptoms are common.^1–3^ Between 20% and 40% of those with CVD have depressive symptoms, which is higher than the prevalence of depression in the general population.^1^ Moreover, depressive symptoms also have negative effects on patients with CVD. Studies have shown that CVD patients with depressive symptoms compared with those without experience poorer health related quality of life and have an increased risk of cardiovascular complications and premature death.^1,3^ Both behavioral and biological mechanisms can explain these negative effects.^2^ Behavioral mechanisms can include lack of treatment adherence,^4^ delay in seeking hospital admission due to a worsening of CVD,^5^ and resistance to performing necessary lifestyle changes.^2^ Biologically, depressive symptoms seem to lead to an increased stress and inflammatory response,^6^ which can lead to worsening of cardiac health.^7^

Having knowledge of which risk factors can lead to depressive symptoms in patients with CVD may be helpful in preventing or detecting such symptoms. Studies have shown that being younger than 60 years or older than 70 years,^8^ being a woman,^8,9^ having severe CVD, or having other previous chronic conditions are such risk factors.^2,8^ However, despite the known risk factors, a high prevalence of depressive symptoms and their negative consequences for CVD, and the fact that European Society of Cardiology Guidelines recommend the treatment of depressive symptoms in patients with CVD,^10^ the recognition rate in cardiac patients is low.^11^ It has been estimated that as few as 15% of CVD patients with depressive symptoms are detected,^12^ and patients with CVD are therefore at risk of not being offered treatment.^13,14^ This is important because the treatment of depressive symptoms in CVD may lead not only to improvements in the symptoms themselves but also to reduced cardiac mortality.^15^

There is limited knowledge as to why the recognition of depressive symptoms is low in patients with CVD. Study authors suggest that factors among healthcare professionals such as lack of time, a focus on medical issues, and no belief in or understanding of the importance treating depressive symptoms can be barriers for not recognizing such symptoms.^16^ Other barriers reported are healthcare professionals' belief that patients are resistant to receiving treatment for depressive symptoms, as well as healthcare professionals not being able to detect these symptoms.^16^ There could also be factors in patients that can act as barriers to recognizing depressive symptoms. Luttik et al^17^ reported that half of CVD patients with depressive symptoms did not want help with treatment of these symptoms,^17^ but the reasons for this were not mentioned. Patients may not clearly express having depressive symptoms in their encounters with healthcare professionals.^16,18^ In a previous study, depressed patients without CVD explained that they did not describe themselves as depressed because of the shame and stigma, fear of a negative response from the environment, and lack of understanding about their depressive symptoms.^19^

Studies focusing on patients' experiences of how depressive symptoms are managed in clinical encounters are scarce,^20^ especially in combination with a somatic chronic illness.^21^ However, a study exploring patients with chronic illness and their beliefs regarding depressive symptoms reported that patients with chronic illness have difficulties in distinguishing between somatic and depressive symptoms, and fear being seen as mentally ill.^22^ This indicates that further work is needed to help us understand the different needs of patients with CVD for the targeting and management of depressive symptoms to facilitate the recognition rate.^22^ By exploring the perspective of patients with CVD, this study aims to contribute to a better understanding of patients' experiences regarding how healthcare professionals in cardiac care address and manage depressive symptoms in a clinical context. Therefore, the aim of this qualitative study was to explore the experiences of patients with CVD of how healthcare professionals address and manage depressive symptoms in clinical cardiac care encounters.

## Methods

This study is a qualitative, semistructured interview study.

### Setting and Participants

Twenty adult patients with CVD from southeastern Sweden were included in this study. To ensure that the research topic was addressed, the participants were recruited from an ongoing randomized controlled trial evaluating an Internet-based cognitive behavior treatment (iCBT) program aimed at reducing depressive symptoms in patients with CVD.^23^ In that trial, patients were included if they had at least mild depression (Patient Health Questionnaire-9 score ≥ 5 points).^24^ Those patients who participated and completed at least 1 treatment module of the iCBT program between January and June 2017 were eligible for inclusion in this interview study. To achieve a broad sample with maximum variation, a purposive sampling method was used. For this thematic analysis, we aimed to include 20 patients. The first 35 participants included in the intervention were invited to participate in this interview study. These potential participants represented a range in terms of sex, age, and type of CVD diagnosis and were contacted through email. Of the 35 invited participants, 20 were willing to participate in this interview study. All participants who had expressed willingness to participate were interviewed. No reasons for not participating were given by those who did not respond. During the final interviews, the authors checked that no new themes were identified, supporting the belief that the variation in the purposive sample had been reached. Characteristics of the participants are presented in Table 1.

**TABLE 1:** Characteristics of Patients Participating in the Study (N = 20)

Characteristics	Frequency (n = 20)	%
Sex		
Female	9	45
Age, y		
Mean (SD)	62 (12)	
Marital status		
Living with partner	17	85
Living alone	3	15
Education		
Elementary	2	10
Upper secondary/high school	7	35
University	11	55
Occupation		
Working	12	60
Retired	8	40
Type of cardiac disease		
Heart failure	1	5
Atrial fibrillation	11	55
Coronary artery/MI/angina	8	40

Abbreviation: MI, myocardial infarction.

This qualitative study conforms with the principles outlined in the Declaration of Helsinki and was approved by the regional ethical review board in Linköping, Sweden (Dnr: 2016/72 31); the iCBT trial is registered at clinicaltrial.org (identifier: NCT02778074). Participants in the iCBT study were informed verbally about the possibility of being contacted to participate in this study and gave written informed consent. Before the interviews were conducted, the participants were informed that they could end the interview at any point during the process without stating a reason. The interviews were recorded with the participants' agreement. All data from the interviews were handled confidentially, and the results are presented in such a way that no individual can be identified.

### Data Collection

Data were collected by means of telephone interviews, which took place between December 2017 and April 2018. The interviews had an average duration of 28 minutes (range, 15–49 minutes). All interviews were conducted by the first author (M.W.), a primary care nurse specialist with previous experience of conducting health assessments by telephone and who is also a PhD student in the iCBT project. The interviewer had no previous relation to the patients and was not involved in their iCBT treatment. To ensure that all the topics of interest were addressed during the interviews, a semistructured interview guide with open-ended questions (Table 2) was used for the purpose of one-to-one interviews.^25^ The interviews started with an introductory question: “Can you tell me about your heart disease?” This was followed by questions about depressive symptoms and patients' experiences of how these were addressed and managed in their encounters with healthcare professionals. Follow-up questions regarding by whom and how depressive symptoms were addressed were asked with the purpose of inviting the participants to elaborate upon their thoughts and experiences. To refine the interview guide to align with the research question, a pilot interview was conducted with one of the participants.

**TABLE 2:** The Interview Guide^a^

Samples of Interview Guide Questions
Introduction
You have been in contact with the health service and been treated for your heart disease. In conjunction with this, you have also been treated for depressive symptoms using our online CBT program.
**Q**uestion 1
1a. Talk a little about your heart disease.
**Q**uestion 2
When you become ill with heart disease, aside from your physical health, your mental health can also be affected. For example, some patients have problems with depressive symptoms after becoming ill.
2a. When you have been in contact with your care provider about your heart disease, have you ever discussed your mental health (eg, depressive symptoms)?
2b. Who brought up the issue of mental health?
2c. What do you think about the information you received that dealt with mental health in cases of heart disease?
2d. What did you do? If nothing, in what way would you have wanted the staff to bring up this issue with you?

Abbreviation: CBT, cognitive behavior treatment.

^a^All interviews were conducted in the participants' native language. The interview guide is translated into English for presentation purposes.

Telephone interviewing has been reported as equally effective as face-to-face interviewing. In collecting data for qualitative research, it has been found to be well accepted by participants and does not affect the final findings.^26,27^ Telephone interviews fit the design of this study because of the geographical locations of the participants, the freedom of accessibility, and the integrity of the participants for whom the program was designed. The dates and times for the interviews were determined by the patients. All interviews were audio-recorded, transcribed, and uploaded into NVivo 12 for analysis.

### Data Analysis

The data were analyzed using a thematic inductive descriptive approach according to Braun and Clarke's^28^ 6 phases to identify, analyze, and report themes within the data. The data were first transcribed into text, read carefully, and verified for accuracy. Then, the transcribed text was coded, starting by generating initial codes in the data. After the initial coding, a search for initial themes was performed, and the first draft of a thematic map was drawn. The initial themes were read again and reviewed in an iterative process against the transcript of the data and study's aim, until the final themes were defined and named.

To ensure the credibility of the analyzed data, triangulation through multiple analysts was conducted in 4 steps. In the first step, 5 randomly chosen transcripts were independently analyzed by the coauthors (M.W., J.L., G.M., M.N., and P.J.). In this step, the coauthors compared their results for selective perceptions and agreed upon the initial themes. In the second step, another 10 transcripts were continually analyzed by all the coauthors in an iterative process, and the themes were reviewed. In the third step, the remaining 5 transcripts were analyzed by the main author. In the last step, all the coauthors discussed, revised, and agreed upon the final themes. Alternative themes and explanations that contradicted the results were tested during the initial and final analysis phases.^29^ The researchers had broad knowledge of CVD, depressive symptoms, and nursing sciences. To ensure the trustworthiness of the study, the authors discussed and defined the aim, methods, and results. Trustworthiness can also be established by transparency in the data analysis, as shown in Table 3.

**TABLE 3:** Example Participant Quotes and Overview of Categories, Subthemes, and Themes of the Analysis

Quotes^a^	Categories	Subthemes	Theme
“It's so transformational and such a big thing to get problems with your heart, because it's still what propels your whole life. Everyone who gets heart problems must get really anxious, but the health service do not dare to talk about, talk about what they cannot cope with listening to, they do not want to hear about how you are feeling.”	Caregivers did not ask about depressive symptoms	The staff did not address my psychological needs	Not being seen as a whole person
“But I remember that I had to fill in a form with some nurse there, about some sort of check-up on how I was feeling. Because I know I felt really bad, because I had so much back pain at the same time for, I'd had it for a long time. That I had not been able to sort out then. And I remember that it was, yes, really bad, in purely physical terms, I remember that. But it was never…it has not been discussed. I cannot remember anyone having asked that.”			
“No, but what I can say is that it was really that what was on offer, it was them, you see, …I guess I'm happy with them. It was, you know, not a question of any therapy really, rather it was someone I would talk to, a conversation, something.”	Feeling of not receiving enough or correct help		
“You get left alone with being ill, you have to just cope with it, it's not interesting for them. They get extremely irritated if you bring up something like that.”	Feeling of having been abandoned by the healthcare system		
“You see, you do not get treated in a way that, yes you have to talk to your GP about that, that's not something we can do much about. Even if they do not come right out any say it, it's like it's…understood.”	Feeling of not being in control of the disease	The staff focused on my somatic symptoms	
“And not just the fact that it's physical things this is about. I would have preferred it if they'd said a bit more.”	Focus on the somatic		
“Nothing, never ever, they have never asked how I'm feeling. I go there and they book an appointment for cardioversion and so you go in and they do they cardioversion and they check that everything is good and then you go home.”			
“No, but it was really relatives who said to me that I should get help because having someone to talk to and someone to talk things through with and what not, you know. But…Then I was…I'm perhaps the kind of person who … Like I did not directly take the initiative to get someone to talk to either.”	Blaming himself for not receiving help	Diminishing and reducing the burden of depressive symptoms	Denying depressive symptoms
“Because I've been really bound up with my illnesses, so I cannot really say that I've been exactly active in talking about them.”	Reducing the problem symptoms of depression		
“No, I really felt that it was more the physical problem that was urgent and that we talked about. And it is highly likely that I tried to hide these mental problems, because I was still at work and had not retired and wanted to be fully committed instead, you know, getting right down to it.”	Explanations to minimize the symptoms		
“As time goes by it's become more, like, has come as thoughts, in situations actually then linked to other things that happened, so these types of reflections have started popping up. And I've, like, gradually started to think about it.”	Late or non–disease awareness	Did not recognize my symptoms as depression	
“Because I felt this wasn't good. You have to get some help. So, making contact with the psychiatric department, actually, I made contact with them myself. And then I got help there, and so I got signed off work and got talking therapy and treatment. So it got sorted out.”	Patient did address the help for depressive symptoms themselves	I was able to communicate my needs	Being provided with help
“It was enough that I was seeing the cardiologist and talking with nurses and such. Because…they offered, uhh…and go there with…I went to one of those heart schools. And there with the cardiologist, like and so then this offer came up of talking to someone as well.”	Take the initiative yourself to get treatment		
“Such a good family, eh! I have a capable wife who's taken care of me, both physically and mentally. I have two wonderful children who have looked after me and this woman whom I'm friends with is a nurse, you see, has changed career a bit, but was originally a nurse, knows everything about these things, she's been other things you know, even so, she's been involved in cancer care and knew about all that stuff. I've got good help there, have not I?”	Working in healthcare Was offered help by cardiac rehabilitation Guided by caregivers Guided by another patient	My social support helped me express my psychological needs	

^a^All quotes were in the participants' native language and then translated into English for presentation purposes.

## Results

In total, 20 patients with CVD (Table 1) participated in the study (9 women; mean age, 62 [range, 34–79] years). Most of the participants were in a relationship (n = 17) and were living in Sweden, in both rural and urban areas.

Three major themes were identified: (1) “not being seen as a whole person,” (2) “denying depressive symptoms,” and (3) “being provided with help.” Each of these 3 major themes has 2 corresponding subthemes (Table 3).

### Not Being Seen as a Whole Person

The first major theme is that the patients felt that they were not seen or identified by healthcare professionals as having any psychological distress and were not treated correctly for their depressive symptoms. The way in which they described having needs that went unidentified and untreated varied from matter-of-factness to anger. Some patients felt that they were emotionally rejected by healthcare professionals when they attempted to initiate a discussion about their depressive symptoms.

#### The Staff Did Not Address My Psychological Needs

Common among the patients was the experience of psychological needs being neglected. The issue of depressive symptoms was either not mentioned at all, only briefly mentioned during the encounter, or presented in a leaflet that patients found in the waiting room. There was a sense that depressive symptoms were not taken seriously or were not part of the treatment during their cardiac care. This led to a feeling of being alone with the depressive symptoms, and despite having received help, patients felt that this help or its quality was not enough to achieve the feeling of having received help.

Everyone who gets heart problems must get really anxious, but the health service does not dare to talk about, talk about what they cannot cope with listening to, they do not want to hear about how you are feeling.

Moreover, patients who were depressed and expressed a need for help with depressive symptoms felt helpless and rejected because their needs were denied or they were told to seek another healthcare professional for their depressive symptoms.

You see, you do not get treated in a way that, yes, you have to talk to your GP about that, that's not something we can do much about. Even if they do not come right out and say it, it's like it's…understood.

#### The Staff Focused on My Somatic Symptoms

A recurring description was that healthcare professionals only focused on the somatic aspects of heart disease and did not see the whole person. Because the focus remained on the somatic factors, the other aspects of having heart disease were not seen or recognized.

And at that time no one knew that it was also having an impact mentally. It was only focused on the physical part. How you were doing, and how you felt and what not, you did not talk about anything else.

### Denying Depressive Symptoms

Patients perceived that they had been in denial concerning their depressive symptoms in previous encounters with healthcare professionals or did not want to reveal their psychological condition.

#### Diminishing and Reducing the Burden of Depressive Symptoms

A recurring perception among patients was that they remembered reducing the severity of their depressive symptoms during the encounter with the healthcare professional, even if they felt a need for help with these symptoms. Many of them blamed themselves for not mentioning depressive symptoms and thus not receiving help. Patients stated that they had difficulties in initiating a discussion about depressive symptoms and sometimes reported suppressing their need for help or guidance.

No, I really felt it was more the physical problem that was urgent and that we talked about. And it’s highly likely that I tried to hide these mental problems, because I was still at work and had not retired and wanted to be fully committed, instead, you know, getting right down to it.

#### Did Not Recognize My Symptoms as Symptoms of Depression

Patients stated that they did not recognize that they had depressive symptoms until later in the treatment process. It was only when enough time had elapsed after the heart event and they had had time to reflect upon their heart disease that they realized they also had depressive symptoms.

As time goes by it's become more, like, has come as thoughts, in situations actually then linked to other things that happened, so these types of reflections have started popping up. And I've, like, gradually started to think about it.

### Being Provided With Help

In this theme, patients did experience being seen and helped by healthcare professionals regarding their depressive symptoms. The experience of getting help for their depressive symptoms depended on either having the ability to communicate their needs regarding these symptoms to their healthcare professionals or having social support to alert them to communicate their needs.

#### I Was Able to Communicate My Needs

Some participants stated that their psychological needs had been met and described how they had been guided toward treatment and the recognition of their depressive symptoms. However, most of those who received help with treatment for depressive symptoms had taken the initiative themselves to start treatment.

Because I felt that this wasn't good. You have to get some help. So, making contact with the psychiatric department, actually, I made contact with them myself. And then I got help there, and so I got signed off work and got talking therapy and treatment. So it got sorted out.

#### My Social Support Helped Me Express My Psychological Needs

Many patients described how guidance from relatives or close friends was a help in addressing depressive symptoms. Most of the social support the patients received came from relatives or close friends who worked in healthcare or had previous personal experience of the healthcare system.

Of those who stated that they had received help for their depressive symptoms, the majority had received guidance or information during cardiac rehabilitation that helped them to recognize depressive symptoms and express a need for help with them.

I went to one of those heart schools. And there with the cardiologist, like and so then this offer came up of talking to someone as well.

## Discussion

To the best of our knowledge, this is one of the first studies to explore the experience of patients with CVD of how depressive symptoms are managed in encounters with cardiac care. We found that CVD patients with depressive symptoms have different experiences and feelings about how these symptoms were addressed by healthcare professionals in cardiac care. Overall, there was a feeling of not being seen as a whole person and that patients with CVD tend to minimize their depressive symptoms, blaming themselves for not asking for help or not showing clear symptoms of depression. Nevertheless, some patients felt that they had received help and treatment.

In this study, patients stated that the issue of depressive symptoms is avoided, both by themselves and by healthcare professionals. Patients experienced that healthcare professionals were mainly focused on the somatic aspects of their heart disease and felt that their psychological needs were not an important part of the CVD treatment and that there was no time to talk about how they felt mentally. One possible explanation for this is that healthcare professionals believed that patients also wanted to focus on their heart disease because this was the primary reason for the clinical encounter. These experiences were described from the subjective perspective of the patients; however, the results of a study investigating primary care physicians' attitudes about the treatment of depressive symptoms in patients with HF or chronic pulmonary disease confirm these experiences. That study reported that common reasons for not offering the patient treatment for depressive symptoms were lack of time and focusing on medical issues.^16^ The experience of not being met as a whole person may lead to the patient developing mistrust of their caregivers, which can result in patients not being comfortable about addressing issues that are experienced as sensitive, such as depressive symptoms. This is important because patients with CVD who report low trust in their healthcare professional are at a higher risk of experiencing worsening of their depressive symptoms.^30^ Furthermore, patients with HF who still have, or have developed, depressive symptoms 18 months after discharge from the hospital are at a higher risk of a worsening prognosis.^31^ This highlights the importance of seeing the patient as a whole and not only as a heart disease.

Another reason for not detecting depressive symptoms is that patients experienced difficulties in addressing these symptoms. Some patients described being aware that they did not feel mentally well but still felt unable to verbalize their state. Some patients stated that they minimized their depressive symptoms during encounters with healthcare professionals and blamed themselves for not clearly expressing them. This can be a sign of being afraid of being stigmatized, which is a common consequence of depression.^32^ Stigma is associated with the belief that they will be perceived as repellent by others within their environment.^19,33^ A study investigating HF patients' views of living with depressive symptoms reported that negative thinking and self-blaming were reasons for not asking for help.^34^ This highlights the importance of healthcare professionals being aware of the stigma of depression or that patients with CVD may have difficulties in understanding that they may have comorbid depressive symptoms. Thus, patients need to learn and understand that depressive symptoms are common in CVD and are not a sign of weakness but could rather be seen as a normal reaction to having a life-threatening disease. Another reason could be due to symptom overlap. Patients reported that, at that point, they did not fully recognize their symptoms as depressive or that they believed their symptoms were part of the heart disease. It has been shown that chronically ill patients, such as those with CVD, can have difficulties in identifying depressive symptoms because these can overlap with somatic symptoms of the CVD.^22,35^ This suggests that healthcare professionals need to be aware of the negative consequences of depressive symptoms in CVD and create a positive clinical care encounter that encourages the patient to reveal and talk about psychological needs.

Although some patients stated that they were able to communicate their depressive symptoms and ask for help in the clinical encounter, this was mostly related to having social support that alerted them to the possibility of doing so, which has also been reported in other CVD studies.^36–38^ Furthermore, having self-confidence and knowledge about depressive symptoms are important factors for addressing and therefore receiving help for such symptoms.^37^ This demonstrates that CVD patients with depressive symptoms who do not have these resources are at risk of not being detected or treated for their depressive symptoms.

## Limitations

A limitation could be that this study only describes personal experiences from the patients' viewpoints and does not capture the issues from all perspectives, which may limit the breadth of the results. Another possible limitation is that the participants may have been biased because of their agreement to participate in the program for treatment of depressive symptoms and thus were possibly not satisfied with the help they had received previously. Furthermore, although we tried to include study participants of different ages, sexes, and CVD diagnoses, we were not able to include a big variation in CVD diagnosis (1 participant with HF, 11 with atrial fibrillation, and 8 with coronary artery/myocardial infarction/angina). For this reason and because the study is conducted in Sweden, the results of this study may not be transferable to all patients with CVD.

## Conclusion

In this study exploring experiences of patients with CVD, they stated that their psychological needs had not been met and that depressive symptoms were overlooked by healthcare professionals in cardiac care. This highlights a need for healthcare professionals to see the patient as a whole to enable the easier detection of depressive symptoms and as an attempt to build trust with the patient to avoid worsening the trajectory of their illness. Patients with CVD who have the ability to address their own needs are better equipped to receive help with depressive symptoms. To strengthen the trust between patients with CVD and caregivers and the patients' own ability to address their needs, healthcare professionals should talk about and assess depressive symptoms and encourage patients with CVD to express emotional problems. More research is needed that focuses on the CVD patient's perspective of having depressive symptoms. In addition, there is a need to explore healthcare professionals' perceptions of how depressive symptoms should be addressed and managed in encounters with patients with CVD.

**What’s New and Important**

- Patients with CVD experience that their psychological needs are neglected and expect healthcare professionals to also discuss depressive symptoms.
- During the encounter with healthcare professionals, patients with CVD either do not reveal their depressive symptoms or reduce the severity of reported symptoms.
- To be recognized as having depressive symptoms and receive treatment for them, patients with CVD need social support or an ability to communicate their needs.
